# Effect of Weighted Vest at 0%, 5% and 10% of Body Mass on Gasometry Biomarkers and Performance during a Rectangular Test in Trained Trail Runners

**DOI:** 10.3390/sports12090229

**Published:** 2024-08-23

**Authors:** Francisco Javier Martínez-Noguera, Pedro E. Alcaraz, Cristian Marín-Pagán

**Affiliations:** Research Center for High-Performance Sport, Campus de los Jerónimos, Catholic University of Murcia, Guadalupe, 30107 Murcia, Spain; palcaraz@ucam.edu (P.E.A.); cmarin@ucam.edu (C.M.-P.)

**Keywords:** endurance exercise, physiology, metabolism, lactate, oxygen saturation, hemoglobin

## Abstract

Trail runners (TRs) must carry an extra load of equipment, food (bars and gels) and liquids, to delay the anticipation of fatigue and dehydration during their competitions. Therefore, we aimed to evaluate how an extra load can influence the metabolic level. Thirteen well-trained trail runners performed a randomized crossover study (total *n* = 39), completing three treadmill running sessions with a weighted vest of 0%, 5% and 10% of their body mass during a combined test (rectangular test + ramp test). In addition, biomarkers of oxygen metabolism, acid–base and electrolyte status pre-, during and post-test, as well as the rectangular from capillary blood of the finger and time to exhaustion, were analyzed. Repeated-measures ANOVA showed no significant difference between conditions for any of the analyzed biomarkers of blood gas. However, one-way ANOVA showed a significant difference in trial duration between conditions (*p* ≤ 0.001). Tukey’s post hoc analysis observed a significant decrease in time to exhaustion in the weighted vest of 10% compared to 0% (*p* ≤ 0.001) and 5% (*p* ≤ 0.01) and 5% compared to 0% (*p* = 0.030). In addition, repeated-measures ANOVA detected a significant difference in pH in the group x time interaction (*p* = 0.035). Our results show that increasing the weighted vest (5% and 10%) anticipates fatigue in runners trained in TR. In addition, increasing the load decreased pH by a smaller magnitude at 10% compared to 0% and 5% at the end of the exercise protocol.

## 1. Introduction

Trail running (TR) is a discipline of sport that takes place in a natural environment such as the mountains and with minimal paved or asphalted roads (<20% of the total duration of the competition) [1]. Due to the increasing popularity of TR, there is a great variety of distances, as we can find races of 20 km (about 1.5 h of competition), 42 km (marathon distance) and long distances (>100 km), although these distances are never fixed and are always combined with large slopes (ascent/descent) [2].

In addition to facing long competition times, trail runners also have to endure rough terrain and adverse weather conditions (such as cold, heat, humidity and altitude) compared to other running disciplines (trail or road), which entails the need to carry a loaded backpack during the competition (safety equipment, nutrition and hydration) [3]. This extra load is justified, as over medium (~42 km) and long (>100 km) distances, a food intake protocol is needed, especially at the ultramarathon distance and especially with several stages, as this type of race has very high minimum food (≥2000 kcal·day^−1^) and fluid requirements (~12 L·day^−1^), which means that runners have to carry a backpack loaded with up to 15 kg (including tent).

This extra load on TRs can lead to metabolic and kinematic imbalances, which can affect race pace and performance compared to an unloaded situation. In this regard, previous studies have shown that an extra weight load (5–30% of body mass (BM)) augments (~5.5%) ground contact time, vertical oscillation (~16.3%) [4,5,6] and leg spring stiffness (~10.5%) [4,5,7]. On the other hand, Purdom et al. [8] evaluated fat oxidation and energy expenditure during an incremental test (60, 65, 70, 75 and 80% VO_2MAX_) with different loads (0%, 5% and 10% of BM) in recreational trail runners. This author observed that with a load of 10% BM, there was a decrease in fat oxidation at an intensity of 70, 75 and 80% VO_2MAX_ in the rectangular test compared to a load of 0% BM. In addition, he also observed an increase in caloric expenditure with a load of 10% BM at an intensity of 65, 70, 75 and 80% VO_2MAX_ compared to a load of 0 and 5% BM [8]. On the other hand, Keren et al. [9] evaluated VO_2_ without load and with load (20 kg) at speeds of 6.4, 7.2, 8.0, 9.6 and 11.2 km/h in active sportsmen students, finding an increase in VO_2_ at the different speeds analyzed with a load of 20 kg on the back compared to without load.

A study has recently been published that evaluated the effect of increasing the load with a weighted vest (0%, 5% and 10% of body mass (BM)) through an incremental test to exhaustion on metabolic, mechanical and performance variables in trained trail runners, where it was observed that increasing the load of 5% (−2.2%) and 10% (−6.1%) BM using a weighted vest produced a loss in performance (they took less time to finish the test) compared to 0% [10]. In addition, they also found that increasing the load of the weighted vest resulted in a loss of speed (5% = −2.2%; 10% = −6.1%) and an increase in absolute power (5% = 2.2%; 10% = 6.1%) at the end of the incremental test compared to 0%. However, in relation to metabolic markers, they found no significant changes in lactate (Lac) and oxygen saturation (sO_2_), but they found a trend in pH in the comparison between conditions. The authors of this article established that an extra load of 5% does not induce noticeable physiological or mechanical changes, but 10% does [10].

Therefore, it is known how an increased extra load affects metabolic and performance markers in trail runners, as these athletes usually carry safety equipment and nutrition. However, it is not known how increasing an extra load specifically affects markers of oxygen metabolism, acid–base status and electrolytes that may explain the recent metabolic and performance changes [10]. Therefore, the main objective of this study is to evaluate the acute effects of running with an extra load (0% vs. 5% vs. 10% of BM) in a rectangular test on biomarkers related to oxygen metabolism, acid–base status and electrolytes (gasometry) utilizing blood gas analysis. We hypothesized that running with an extra load of 5% and 10% BM significantly decreases time to exhaustion in a combined test, a decrease in oxygen saturation and pH, and an increase in lactate. The data in this paper are part of a project where, due to the density of the data collected, they have been published in several papers, with secondary data appearing in the current paper.

## 2. Materials and Methods

### 2.1. Study Design

This study utilized a randomized, crossover experimental design. To allocate conditions, a software called Randomizer was employed to assign codes [11]. Well-trained trail runners participated and completed a combined test involving a rectangular test and a ramp test under three different conditions: 0% (control; 0% BM load), 5% (5% BM load) and 10% (10% BM load). Biomarkers of oxygen metabolism, acid–base status and electrolytes were examined using finger capillary blood gasometry before, during and after the exercise.

### 2.2. Participants

The study involved thirteen men who were amateur trail runners. You can find the details about the athletes in Table 1. The criteria for inclusion were as follows: age between 18 and 35 years, BMI of 19.0–25.5 kg·m^−2^, a minimum of three years of experience in trail running and 6–12 h of training per week (at least 4 sessions per week). Participants were excluded if they (a) were smokers or regular alcohol consumers; (b) had metabolic, cardiorespiratory or digestive health issues; (c) had experienced an injury in the last six months; (d) had taken any supplements or medication in the previous two weeks; and (e) had abnormal values for any baseline health blood test parameter. The sample size was determined using G*POWER 3.1.9.7 software (University of Düsseldorf, Düsseldorf, Germany). The setup used for this purpose was: F-ANOVA test: repeated measures, within-between interaction; A priori. Effect size f = 0.5; α error prob = 0.05; power (1-β error prob = 0.80). The outcome indicated an appropriate total sample size of 12 subjects. The eligible participants provided informed consent before participating in the study. This study adhered to the guidelines of the Declaration of Helsinki on Human Research [12] and was approved by the Ethics Committee of the university (CE012104). All the participants successfully completed the study.

### 2.3. Procedures

The TR runners visited the laboratory five times at a minimum interval of 6 days. On the first visit, participants were informed about the procedures and tests to be performed during the study, and a medical examination was performed to check their health status. In addition, body composition was assessed by anthropometry. Participants were informed that they should follow a set diet (by a nutritionist) at all test visits and that they should not train in the previous 24 h. At the second visit, familiarization of the combined test (rectangular test + ramp test) was performed with a random load (0, 5 or 10% BM). On visits 3, 4 and 5, a combined test (rectangular test + ramp test) with the different loads (0, 5 and 10% BM) was performed in random order. The mean of the 5% condition was 3.12 kg and the mean of the 10% condition was 6.25 kg. All participants completed the study (total *n* = 39). In the 24 h prior, no training could be performed, and in the 72 h prior, only low–moderate-intensity training could be performed. On the days before visits 3, 4 and 5, participants consumed a standardized diet consisting of 0.9 g/BM fat, 1.5 g/BM protein and 9.0 g/BM carbohydrate. In addition, athletes were instructed to eat a standardized breakfast 2 h before the tests, consisting of 0.43 g/BM protein, 1.3 g/BM carbohydrate and 0.57 g/BM fat. The previous day’s meals and pre-test breakfasts were prepared by a sports nutritionist and sent to participants 2 weeks before the start of the study.

### 2.4. Tests

#### 2.4.1. Medical Exam

The health assessment included a review of the participant’s medical history, a resting electrocardiogram and an examination by a medical doctor consisting of auscultation and measuring blood pressure to ensure the participants’ eligibility for the study based on their health.

#### 2.4.2. Blood Samples

A certified nurse performed the extraction of venous blood, obtaining one 3 mL tube of ethylenediaminetetraacetic acid (EDTA) for hemogram and another 3.5 mL tube with polyethene terephthalate (PET) for health analysis. An automated Cell-Dyn 3700 analyzer (Abbott Diagnostics, Chicago, IL, USA) was used to conduct a red blood cell count, with internal (Cell-Dyn 22) and external (Program of Excellence for Medical Laboratories-PEML) controls being utilized. The analyzer estimated values of erythrocytes, hemoglobin, hematocrit and hematimetric indexes.

#### 2.4.3. Anthropometry

A researcher certified at ISAK Level 1 conducted the anthropometric measurements (FJMN). A digital scale with a stadiometer for clinical use (SECA 780; Vogel & Halke GmbH & Co., Hamburg, Germany) was used to measure height and body weight. Skinfold thickness was assessed with Holtain Skinfold Calipers (Holtain, Ltd., Crymych Pembrokeshire, UK) in adherence to the guidelines of the International Society for the Advancement of Kinanthropometry [13]. Body fat percentage was determined using the Faulkner equation [14], and muscle mass percentage was calculated using the modified Matiegka equation [15]. The total of the eight skinfolds (triceps, subscapular, bicep, iliac crest, supraspinal, abdominal, thigh and calf) was also computed.

#### 2.4.4. Familiarization and Incremental Test Protocol

A combined test (rectangular test + ramp test) was performed as a two-phase protocol on a treadmill (Technogym Excite Med. Cesena, Italy). This combined method was used to assess ventilatory thresholds 1 and 2 (VT1 and VT2) with a step phase, followed by a ramp phase to assess peak value [16,17,18]. Step phase started at 5 km/h and velocity increased by steps of 1 km/h every 2 min. This first phase ended when RER reached a value of 1.00 for more than 30s (25% of the total step time) in the same step (POST1). At this point, athletes were asked to indicate their effort perception on a modified Borg Scale (RPE) from 1 to 10 [19]. Participants then proceeded to rest for 5 min in standing position. The objective of this resting period was to ensure the maximality of the second phase by reducing fatigue associated with the first one [20]. Second phase started at final first-phase velocity and increased 1.5 km/h each min as a ramp test (0.15 km/h every 3 s), ending at exhaustion (POST2). Athletes again indicated RPE at this point.

#### 2.4.5. Blood Gas Analysis (ABL-90)

The levels of oxygen metabolism, acid–base status and electrolyte biomarkers were assessed by analyzing arterialized capillary blood from the fingertip while at rest (PRE), at the conclusion of the initial phase (POST1) and after the exercise protocol (POST2). Hematocrit (Hct), hemoglobin (Hb), O_2_ partial pressure (pO_2_), carbon dioxide (pCO_2_), O_2_ pressure (sO_2_), oxyhemoglobin (O_2_Hb), carboxy-hemoglobin (COHb), deoxyhemoglobin (RHb), methemoglobin (MetHb), total blood O_2_ concentration (tO_2_), total blood carbon dioxide concentration (tCO_2_), O_2_ partial pressure at 50% oxygen saturation (p50), non-oxygenated blood fraction (Shunt) and the difference between the alveolar concentration (A) of O_2_ and the arterial (a) concentration of O_2_ (AaDpO_2_) were all determined. The ABL 90 FLEX blood gas analyzer (Radiometer Medical ApS, Copenhagen, Denmark) was utilized to measure these parameters and underwent calibration at hourly intervals throughout the day using internal reference standards. Previous research has indicated that the ABL90 FLEX provides accurate results [21]. Plastic capillary tubes were pre-heparinized with electrolytically balanced solid heparin, significantly reducing clotting risk and ensuring unbiased and reliable electrolyte results.

#### 2.4.6. Statistical Analysis

The statistical analysis was conducted using IBM Social Sciences software (SPSS, v.21.0, Chicago, IL, USA). Mean ± SD were used to present the data. The Levene and Shapiro–Wilk tests were employed to check the homogeneity and normality of the data, respectively. A two-way repeated-measures ANOVA was used to analyze each gasometry biomarker, with factors including time (PRE vs. POST1 vs. POST2) and condition (0% vs. 5% vs. 10% BM). A one-way ANOVA was performed for the trial duration. In the case of significance in the ANOVA models, Tukey’s post hoc analysis was carried out. Partial eta squared (ηp^2^) was also calculated as an effect size for time, condition and time × condition interaction of all variables in the ANOVA analysis. The thresholds for partial eta squared were applied as follows: <0.01, irrelevant; ≥0.01, small; ≥0.059, moderate; ≥0.138, large [22]. The significance level was set at *p* ≤ 0.05. Pearson’s correlation (r) was used to evaluate the correlations between the parameters.

## 3. Results

**Figure 1:**
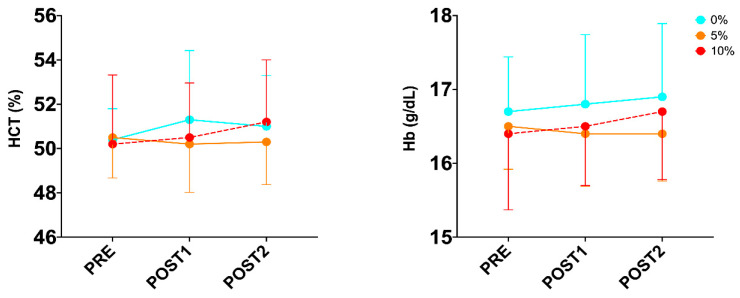

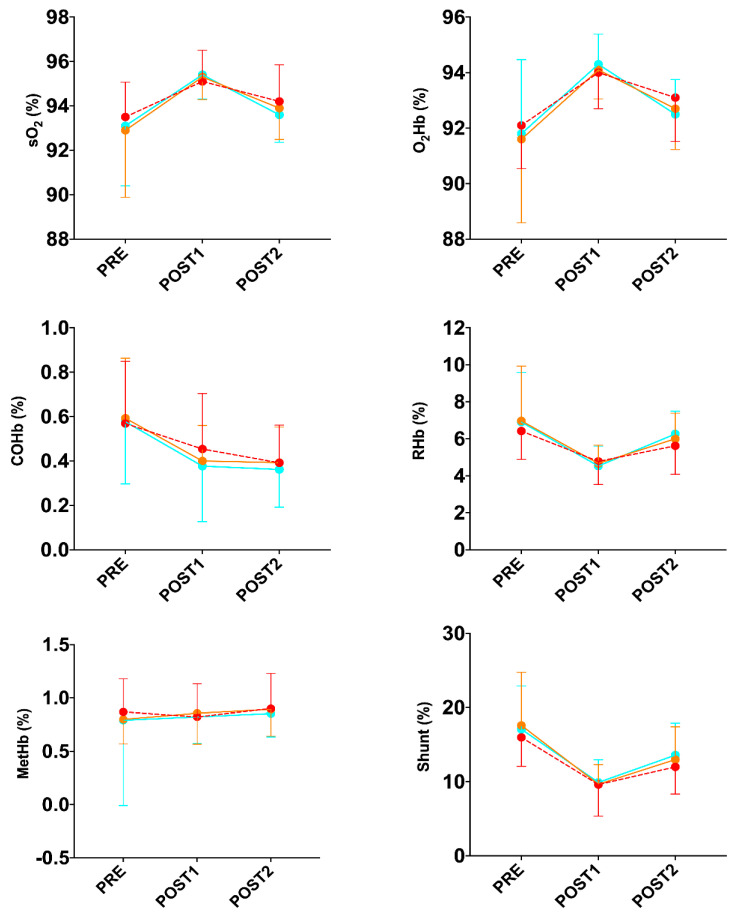
Changes in biomarkers of oxygen metabolism during exercise protocol in well-trained trail runners.

**Figure 2:**
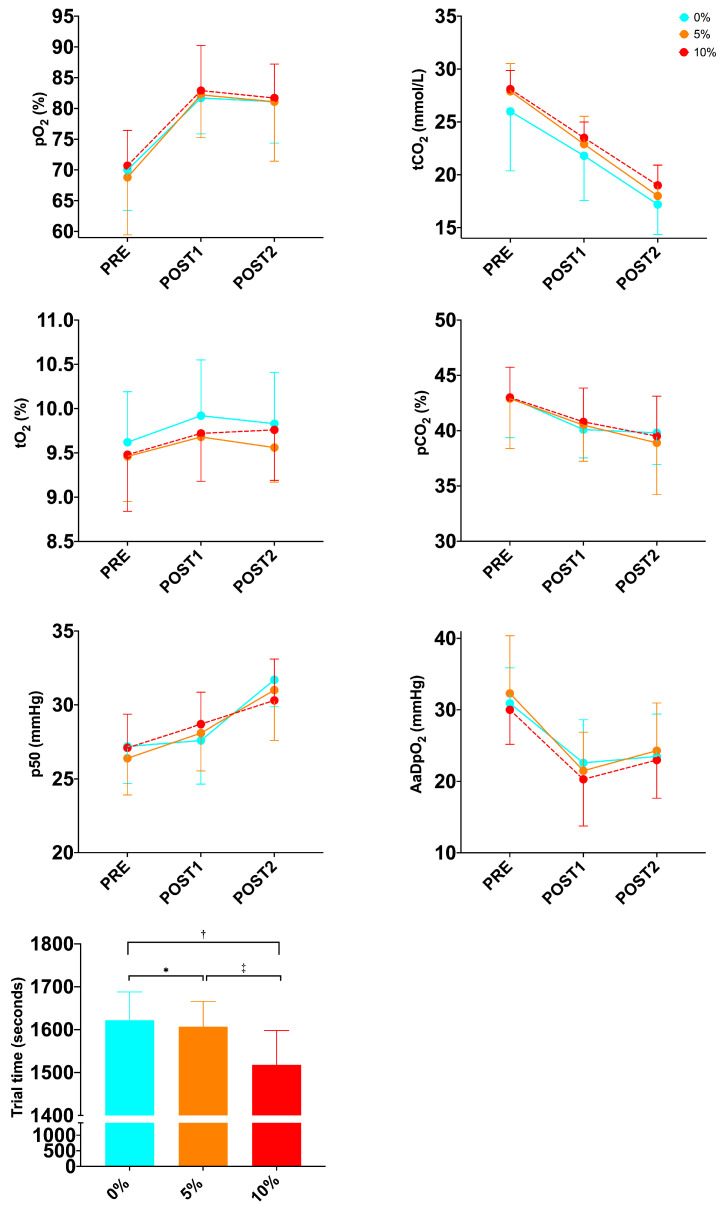
Changes in biomarkers of oxygen metabolism and trial time during exercise protocol in well-trained trail runners. *—*p* = 0.03 between 0% and 5%; ^†^—*p* ≤ 0.001 between 0% and 10% and ^‡^—*p* ≤ 0.01 between 5% and 10%.

After performing the two-way repeated-measures ANOVA, no significant difference was observed in the condition x time interaction for biomarkers of oxygen metabolism (Table 2; Figure 1 and Figure 2), but a trend was observed in the MetHb (*p* = 0.064; ηp^2^ = 0.166). On the other hand, when analyzing the differences in time to exhaustion between the three conditions during the combined test, one-way ANOVA detected a condition main effect (*p* ≤ 0.001; ηp^2^ = 0.641). Tukey’s post hoc analysis showed a significant difference between 0% and 5% (0.029), between 0% and 10% (*p* ≤ 0.001) and between 5% and 10% (*p* = 0.003).

**Figure 3:**
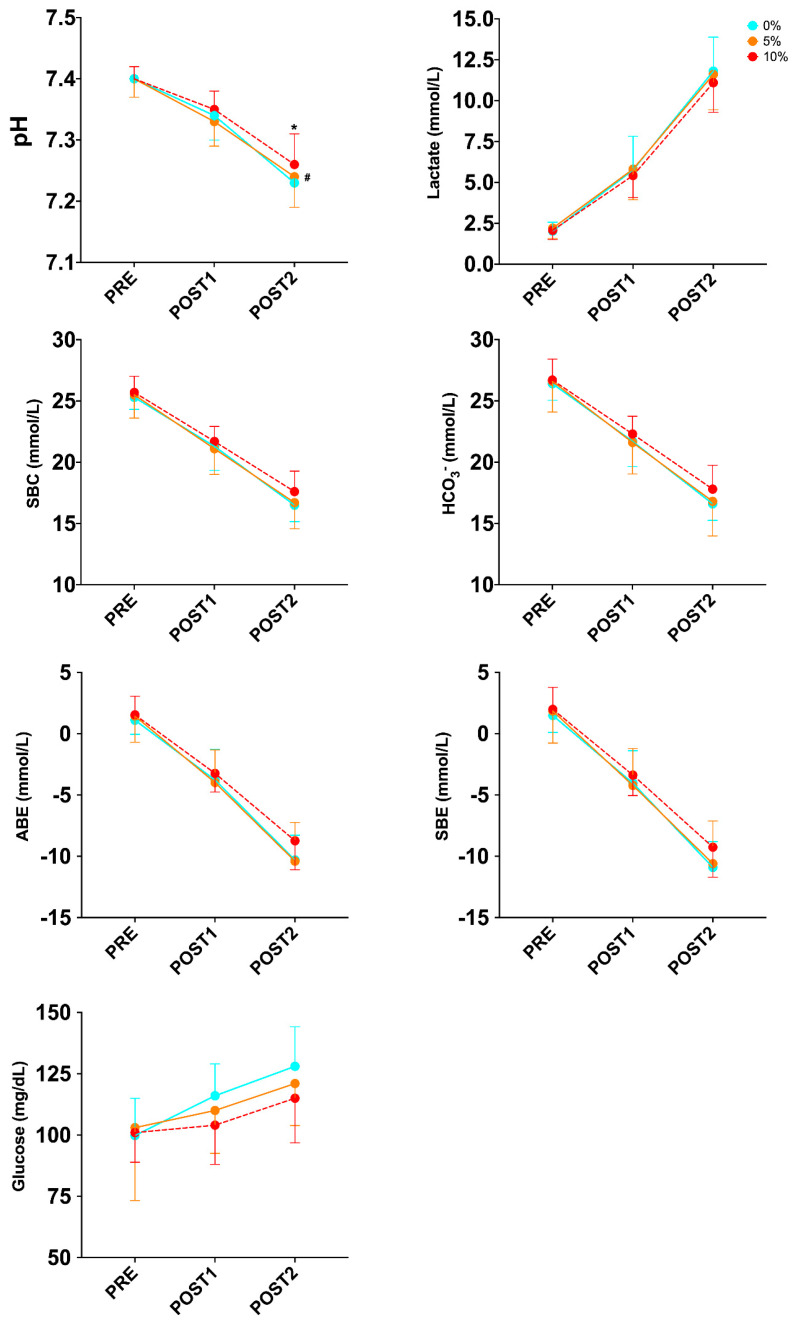
Changes in biomarkers of acid–base status and glycemia during exercise protocol in well-trained trail runners. *—significant difference (*p* ≤ 0.050) between 0% and 10%; ^#^—trend (*p* = 0.082) between 5% and 10%.

For the variables related to acid–base status and glucose (Table 3), the two-way ANOVA with repeated measures detected no significant change in the condition x time interaction, except for pH (*p* = 0.035; ηp^2^ = 0.191). Tukey’s post hoc analysis showed a significant difference in pH between 0% and 10% (*p* = 0.004) and a trend between 5% and 10% (*p* = 0.082) in POST2. (Figure 3). This indicates that a weighted vest with 10% BM produces a smaller decrease in pH than with 0% and 5% BM at the end of the combined test. Despite the different loads used during the exercise protocol, no significant change was observed in the condition x time interaction in Lac (*p* = 0.775).

**Figure 4:**
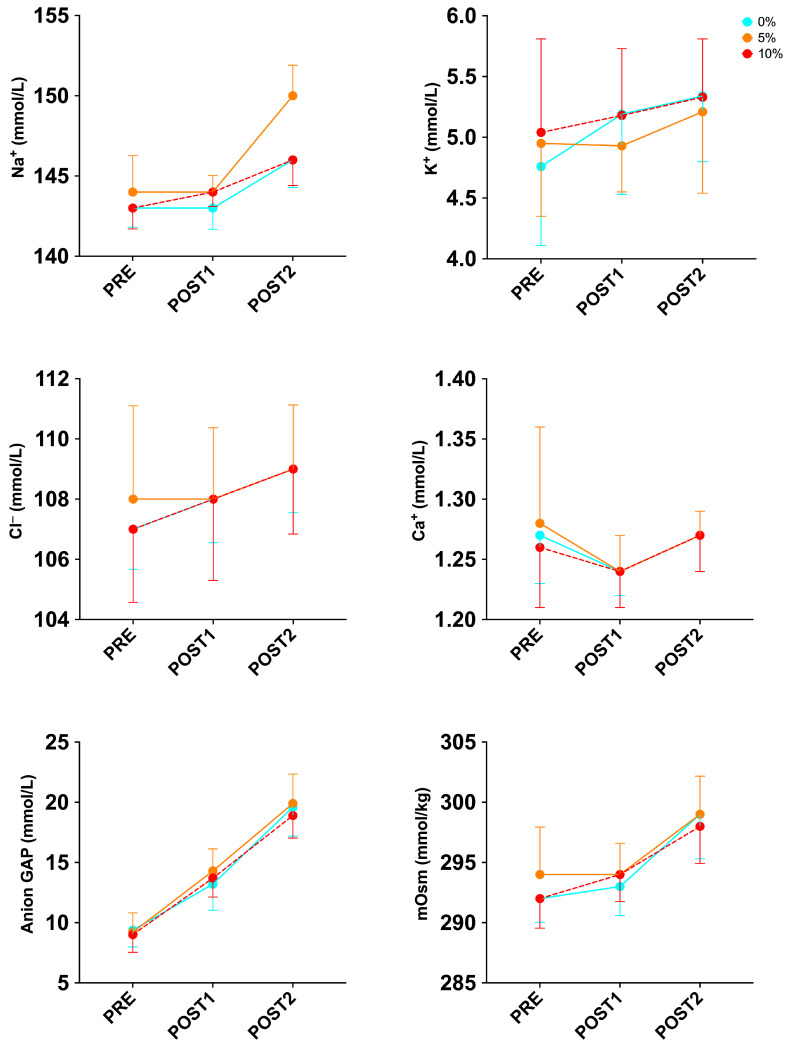
Changes in electrolyte biomarkers and osmolarity during exercise protocol in well-trained trail runners.

Finally, within the biomarkers of electrolytes and osmolarity, after performing two-way repeated-measures ANOVA, we found no significance in the condition x time interaction (Figure 4; Table 4).

**Figure 5:**
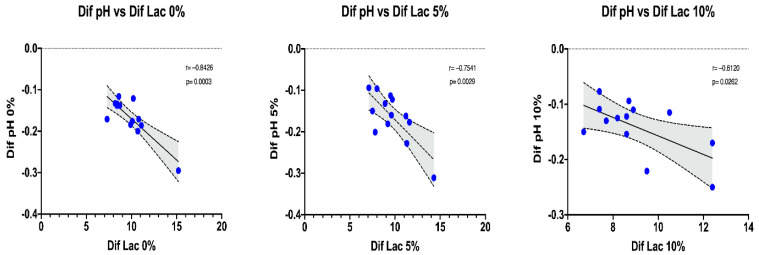
Correlations of pre–post exercise protocol differences between pH and lactate in well-trained trail runners.

Furthermore, we analyzed the correlations of the pre–post differences of the different intervention conditions (0, 5 and 10% BM) between pH and Lac. In Figure 5, we can observe that the correlations between the pre–post differences between pH and Lac are strongest at 0% (r = −0.8426; *p* = 0.0003) and 5% (r = −0.7541; *p* = 0.0029) loading but weakest at 10% loading (r = −0.6120; *p* = 0.0262).

## 4. Discussion

To our knowledge, this is the first study that has examined how increasing the load using a weighted vest (0% vs. 5% vs. 10% BM) affects biomarkers of oxygen metabolism, acid–base status and electrolytes during a combined test in trained trail runners. After evaluation of the different loads during the exercise protocol, it was found that a 10% weighted vest load anticipated fatigue compared to 0% and 5% BM. In addition, pH was higher with a 10% load compared to 0% and 5% at the end of the exercise protocol, indicating lower metabolic stress with a 10% BM weighted vest load at the end of the exercise protocol.

One of the main findings of this study was the reduction in exercise time (loss of performance) by increasing the weighted vest load to 10% BM (1989 s). This situation anticipated fatigue in the exercise protocol compared to 0% (2117 s) and 5% (2065 s). In line with our results, a recent study also showed a decrease in time to exhaustion (decrease in performance) in an incremental test with the addition of an extra load using a weighted vest (0% vs. 5% vs. 10%) in trained trail runners [10]. On the other hand, the effect of a weighted vest (0%, 5% and 10% BM) on kinematic variables at different running speeds (8, 10, 12 and 14 km·h^−1^) has been evaluated in trail runners [23]. This author found that running with 10% BM decreased flight time and increased duty factor and ground contact time at speeds of 8, 10, 12 and 14 km·h^−1^ compared to 0% and 5% BM loading. On the other hand, one study tested how different loads of 10, 20 and 30% BM at a constant speed (10K pace; 3.34 ± 0.22 m·s^−1^) can affect the kinematic pattern of running in recreational trail runners [4]. It has been described that carrying loads causes modifications in gait kinematics [23,24]. Moreover, the addition of a load during running leads to decreased stride length, increased cadence and pelvic tilt and hip flexion [25,26]. However, it has been shown that the load of extra weight can modify ground reaction forces, with increased vertical ground reaction forces being proportional to the amount of load carried [25,26,27,28,29,30]. Moreover, extra loading leads to a large increase in the rate of soil reaction loading [30].

In addition, it should be noted that skeletal muscles play an important role in external load attenuation. During the loading response of running and landing in a fall, leg muscles contract eccentrically to reduce impact forces against the ground [31]. However, muscle fatigue reduces force production and the muscle’s ability to reduce ground impact forces [32,33]. Moreover, it has been shown that there is a modification in running kinematics associated with muscle fatigue [34,35,36]. Precisely, the knee joint becomes stiff with less flexion during weight acceptance [35]; the ankle joint shows less dorsiflexion at heel contact [36]. Specifically, muscle fatigue results in increased vertical ground reaction forces and ground reaction loading rates during landing [37], running [36] and walking [30]. Therefore, according to our results, the performance loss as a consequence of an increase in the weighted vest (0% vs. 5%: −2.5%; 0% vs. 10%: −6.0%; 5% vs. 10%: −3.7%) during the exercise protocol may be due to the combined effect of extra load carrying coupled with changes in joint kinematics and kinetics as a consequence of muscle fatigue.

Furthermore, this loss of performance in the incremental test with increasing load found in our study also coincides with results found by Jiménez-Redondo et al. [10], where they observed a significant decrease in peak velocity at 10% BM (−6.6%) but not at 5% (−2.0%) compared to 0%. On the other hand, they also found that increasing the load resulted in a higher maximum absolute power output (0% = 374 ± 60 W; 5% = 382 ± 59 W; 10% = 397 ± 58 W). Furthermore, at ventilatory threshold 1, they observed a decrease in velocity (−10.3%) and an increase in running economy (14.4%) at 10% BM load compared to 0% [10]. In the same study, they also found a decrease in velocity at ventilatory threshold 2 when comparing a 10% BM load to 0%. Therefore, it can be established that the increase of an extra 10% BM load employing a weighted vest can negatively affect performance and mechanical variables, which can offer valuable information to trail runners and coaches to ensure optimal management of the extra load that runners should carry in their competitions.

The effect of walking speed (0.89, 1.12, 1.34, 1.56 and 1.79 m·s^−1^) combined with an extra load (0%, 10%, 15% and 20% BM) on indirect calorimetry variables has been evaluated [38]. For the four weighted vest conditions, the authors observed a classic curvilinear relationship between oxygen consumption and walking speed, finding a significant interaction (*p* = 0.001) between speed and weighted vest conditions. The authors found that as speed increased, there were greater differences in oxygen consumption between the weighted vest and speed conditions [38]. Moreover, another author evaluated how a weighted vest affects men (9.07 kg; 11.8% BM) and women (6.35 kg; 10.0% BM) during a 30 min running test at 55% VO_2MAX_ on physiological–metabolic variables in university-level athletes [39]. During the exercise protocol, the inclusion of a weighted vest increased VO_2_ (men: +0.22; women: +0.07 L·min^−1^), VE/VO_2_ (men: 7.2%; women: 5.7%) and heart rate (men: 7%; women: 7%) in both sexes. In addition, changes in substrate utilization were also found, as the application of the weighted vest increased carbohydrate oxidation (men: +0.6; women: +0.2 g/min) and decreased fat oxidation (men: −0.18; women: −0.06 g/min), the changes observed in women being 33% of the change in substrate utilization observed in men [39]. These shifts in substrate oxidation were coupled with changes in Lac at the end of the exercise protocol but only in men (men: +0.6; women: +0.1 mmol/L) [39].

However, no significant change was found in metabolic markers in capillary blood between conditions, except for pH, with no change in Lac. As indicated in Figure 3, the upward kinetics in Lac was similar in the three intervention conditions in POST1 (0%: 5.8; 5%: 5.8; 10%: 5.4 mmol/L) and POST2 (0%: 11.8; 5%: 11.6; 10%: 11.1 mmol/L). In addition, significant changes in pH were mainly between the 0% and 10% BM weighted vest, with a trend between 5% and 10%, indicating a greater decrease in pH at 0% and 5% loading than at 10% loading. Although there are differences between our exercise protocol and the one used by Gaffney et al. [39], since, in our case, the aim was to assess metabolic variables with different weighted waistcoats and intensities, the aim of Gaffney et al. was to assess metabolic variables during a defined intensity and time. These differences in exercise protocol may be key to finding differences in metabolic biomarkers.

It is known that during an incremental test with increasing intensity, there is an inverse relationship between Lac and pH. For example, an increase was observed in Lac from rest to FatMax (65%), VT1 (27%), VT2 (390%) and maximum power (767%), with decreasing pH and HCO_3_^−^ in finger capillary blood during a rectangular test but with increasing intensity in amateur cyclists [40]. It is known that VT2 is an exercise zone where metabolic acidosis is high but stable, but when this zone is exceeded, a fatigue-inducing accumulation of metabolites (↑ lactic acid and H^+^) [41] and changes in the recruitment pattern of muscle-activating motor units are generated [42]. In addition, the accumulation in the muscle cell of H^+^ may also promote the development of fatigue by reducing the glycolytic rate and has been shown to act synergistically with inorganic phosphate (Pi) to compromise cross-bridge function [43]. In this regard, a recent study concluded that Pi is the primary cause of peripheral fatigue and muscle acidosis, probably acting on condition III/IV muscle afferents in the interstitial space, as a contributing factor to central fatigue during exercise [44]. During submaximal steady-state exercise, Lac production (input) is equal to Lac washout (output), where Lac concentration remains constant [45]. But at exercise intensities above steady state, an increase in concentration could be due to an increase in the rate of Lac generation or a decrease in the rate of clearance [45].

Decreased pH in muscle fibers can produce changes in muscle metabolism, leading to changes in neuromuscular and kinematic patterning, resulting in a loss of performance, without changes in Lac. Therefore, we can state that an increase in load in a weighted vest produces a smaller decrease in pH, with no change in Lac between conditions, but with a greater loss of performance with an increase in load (0% vs. 5% vs. 10% BM). These data indicate that fatigue has a high metabolic component for 0% and 5% weighted vests but with a lower metabolic component and higher neuromuscular component with 10% BM loading. This is in line with the results found in the correlations between pH and Lac differences pre–post exercise protocol, where they were stronger with a 0% and 5% BM weighted vest load (r = −0.8426, r = −0.7541, respectively) than with 10% (r = −0.6120). For future studies, in addition to measuring these markers pre- and post-exercise, we believe it would be very interesting to evaluate the acid–base status variables in metabolic zones such as FatMax, VT1 and VT2 during a step test and see if there is a shift in these zones concerning time and speed with an increase in load vest.

Therefore, it is possible to state that an increase in load (0% vs. 5% vs. 10% BM) in a weighted vest produces a loss of performance (reduction in time to exhaustion) in a combined test with a smaller decrease in pH, with no change in Lac between conditions. The greatest negative effects on pH occurred with the 0% BM weighted vest, indicating that there was greater metabolic stress in this condition. However, with a 10% BM weighted vest, the pH does not decrease as much as the other conditions (0% and 5%), probably because neuromuscular fatigue factors are involved [23], which do not allow the maximum metabolic potential to be expressed at this load.

Admittedly, there are some limitations to this study that need to be mentioned. We included trained male trail runners in this study, so we do not know what the results found in this study would be in high-performance athletes or of another gender. Another limitation of the study is that arterialized capillary blood from the fingertip is a mixture of arterial and venous blood, and for some biomarkers, their correspondence is weak between the results of capillary and arterial blood samples. When performing the pre-, during and post-test combined blood gas measurements, we do not know how the variables assessed during the test evolve. Furthermore, the evaluations were carried out in a laboratory with a combined test (divided into two phases), and this type of exercise does not fit the reality of trail running competitions, neither in terms of duration nor intensity. Therefore, there is a need for further research to generate a physiological–biochemical characterization of the TR, especially in real competition conditions and to find out how an increase in additional loads can affect performance. Future research should include high-performance athletes, female athletes and a more diverse sample to understand the broader applicability of the findings. Conducting experiments under conditions that resemble actual trail running competitions, with similar durations and intensities, would yield more relevant and applicable results. Thus, future research using portable biomarker monitoring equipment during real trail runs could be the ideal study design to generate the physiological–metabolic profile based on the extra load carried.

## 5. Conclusions

Our results show that increasing the load of the weighted vest (0%, 5% and 10% BM) produces an exponential loss of performance during a combined test in trained TRs. Furthermore, increasing the load produces a smaller decrease in pH, with no change in Lac or any other metabolic marker in capillary blood. These findings allow us to affirm that the anticipation of fatigue with a 10% BM weighted vest has a greater neuromuscular than metabolic component compared to 0% and 5% BM.

## Figures and Tables

**Table 1:** General characteristics of TR runners. Mean (standard deviation (SD)).

Age (years)	28.0 (8.7)
Body mass (kg)	62.5 (3.8)
Height (cm)	173.3 (0.5)
Distance/week (km)	58.8 (2.5)
Elevation gain/week (m)	1662.5 (110.9)
Fat mass (%)	9.3 (0.7)

**Table 2:** Changes in biomarkers of oxygen metabolism in capillary blood of the finger in pre, post1 and post2 incremental test. Values are mean (SD).

		0%	5%	10%	Time	Condition	C × T
Hematocrit (%) (HCT)	Pre	50.4 (1.40)	50.5 (1.83)	50.2 (3.12)	*p* = 0.261	*p* = 0.020	*p* = 0.712
	Post1	51.3 (3.12)	50.2 (2.18)	50.5 (2.46)			
	Post2	51.0 (2.30)	50.3 (1.92)	51.2 (2.80)			
	ηp^2^				0.106	0.279	0.051
Hemoglobin (g/dL) (Hb)	Pre	16.7 (0.74)	16.5 (0.58)	16.4 (1.03)	*p* = 0.261	*p* = 0.020	*p* = 0.825
	Post1	16.8 (0.94)	16.4 (0.71)	16.5 (0.80)			
	Post2	16.9 (0.99)	16.4 (0.64)	16.7 (0.92)			
	ηp^2^				0.106	0.279	0.030
Oxyhemoglobin (%) (O_2_Hb)	Pre	91.8 (2.67)	91.6 (3.01)	92.1 (1.56)	*p* ≤ 0.001	*p* = 0.596	*p* = 0.814
	Post1	94.3 (1.09)	94.1 (1.05)	94.0 (1.30)			
	Post2	92.5 (1.25)	92.7 (1.47)	93.1 (1.58)			
	ηp^2^				0.507	0.042	0.032
Carboxy-hemoglobin (%) (COHb)	Pre	0.577 (0.28)	0.593 (0.27)	0.569 (0.28)	*p* ≤ 0.001	*p* = 0.315	*p* = 0.505
	Post1	0.377 (0.25)	0.400 (0.16)	0.454 (0.25)			
	Post2	0.362 (0.17)	0.393 (0.16)	0.392 (0.17)			
	ηp^2^				0.519	0.092	0.066
Deoxyhemoglobin (%) (RHb)	Pre	6.81 (2.68)	6.97 (2.96)	6.42 (1.53)	*p* ≤ 0.001	*p* = 0.496	*p* = 0.826
	Post1	4.53 (1.07)	4.67 (0.99)	4.78 (1.25)			
	Post2	6.26 (1.23)	6.00 (1.39)	5.62 (1.54)			
	ηp^2^				0.463	0.057	0.030
Methemoglobin (%) (MetHb)	Pre	0.792 (0.80)	0.800 (0.23)	0.869 (0.31)	*p* = 0.003	*p* = 0.487	*p* = 0.064
	Post1	0.823 (0.25)	0.857 (0.29)	0.823 (0.31)			
	Post2	0.854 (0.22)	0.893 (0.25)	0.900 (0.33)			
	ηp^2^				0.378	0.058	0.166
Oxygen saturation (%) (sO_2_)	Pre	93.1 (2.70)	92.9 (3.02)	93.5 (1.57)	*p* ≤ 0.001	*p* = 0.655	*p* = 0.825
	Post1	95.4 (1.10)	95.3 (1.02)	95.1 (1.41)			
	Post2	93.6 (1.24)	93.9 (1.41)	94.2 (1.65)			
	ηp^2^				0.460	0.035	0.030
Oxygen partial pressure (mmHg) (pO_2_)	Pre	70.0 (6.57)	68.8 (9.36)	70.7 (5.73)	*p* ≤ 0.001	*p* = 0.678	*p* = 1.000
	Post1	81.7 (5.82)	82.2 (6.95)	82.9 (7.35)			
	Post2	81.1 (6.74)	81.1 (9.67)	81.7 (5.52)			
	ηp^2^				0.840	0.032	0.001
Carbon dioxide partial pressure (mmHg) (pCO_2_)	Pre	43.0 (3.63)	42.9 (4.51)	43.0 (2.73)	*p* ≤ 0.001	*p* = 0.964	*p* = 0.704
	Post1	40.1 (2.56)	40.5 (3.27)	40.8 (3.05)			
	Post2	39.8 (2.86)	38.9 (4.68)	39.5 (3.62)			
	ηp^2^				0.552	0.003	0.043
Total blood oxygen concentration (mmol/L) (tO_2_)	Pre	9.62 (0.57)	9.46 (0.51)	9.48 (0.64)	*p* = 0.007	*p* = 0.007	*p* = 0.752
	Post1	9.92 (0.63)	9.68 (0.50)	9.72 (0.54)			
	Post2	9.83 (0.58)	9.56 (0.39)	9.76 (0.57)			
	ηp^2^				0.335	0.341	0.038
Total blood carbon dioxide concentration (mmol/L) (tCO_2_)	Pre	26.0 (5.62)	27.9 (2.63)	28.1 (1.77)	*p* ≤ 0.001	*p* = 0.214	*p* = 0.693
	Post1	21.8 (4.22)	22.9 (2.64)	23.5 (1.49)			
	Post2	17.2 (2.84)	18.0 (2.93)	19.0 (1.91)			
	ηp^2^				0.977	0.143	0.053
Oxygen partial pressure at 50% oxygen saturation (mmHg) (p50)	Pre	27.2 (2.51)	26.4 (2.49)	27.1 (2.27)	*p* ≤ 0.001	*p* = 0.773	*p* = 0.136
	Post1	27.6 (2.96)	28.1 (2.56)	28.7 (2.16)			
	Post2	31.7 (1.82)	31.0 (3.40)	30.3 (2.80)			
	ηp^2^				0.639	0.021	0.133
Relative physiological Shunt (%) (Shunt)	Pre	17.1 (5.80)	17.6 (7.15)	16.0 (3.92)	*p* ≤ 0.001	*p* = 0.491	*p* = 0.987
	Post1	9.91 (3.07)	9.66 (2.66)	9.62 (4.26)			
	Post2	13.6 (4.32)	13.0 (4.42)	12.0 (3.66)			
	ηp^2^				0.663	0.058	0.007
Alveolar–arterial gradient (mmHg) (AaDpO_2_)	Pre	30.9 (4.98)	32.3 (8.08)	30.0 (4.80)	*p* ≤ 0.001	*p* = 0.531	*p* = 0.980
	Post1	22.6 (6.01)	21.5 (5.39)	20.3 (6.55)			
	Post2	23.5 (5.90)	24.3 (6.65)	23.0 (5.35)			
	ηp^2^				0.705	0.051	0.009
Trial duration (seconds)		2117 *^†^ (134)	2065 ^‡^ (145)	1989 (157)			*p* ≤ 0.001
	ηp^2^						0.641

*—*p* = 0.03 between 0% and 5%; ^†^—*p* ≤ 0.001 between 0% and 10% and ^‡^—*p* ≤ 0.01 between 5% and 10%.

**Table 3:** Changes in biomarkers of acid–base state and glucose in capillary blood of the finger in pre, post1 and post2 incremental test. Values are mean (SD).

		0%	5%	10%	Time	Condition	C × T
pH	Pre	7.40 (0.03)	7.40 (0.03)	7.40 (0.02)	*p* ≤ 0.001	*p* = 0.030	*p* = 0.035
	Post1	7.34 (0.04)	7.33 (0.04)	7.35 (0.03)			
	Post2	7.23 (0.04) *	7.24 (0.05) ^#^	7.26 (0.05)			
	ηp^2^				0.897	0.253	0.191
Lactate (mmol/L) (Lac)	Pre	2.02 (0.55)	2.20 (0.62)	2.07 (0.57)	*p* ≤ 0.001	*p* = 0.405	*p* = 0.775
	Post1	5.76 (2.06)	5.81 (1.87)	5.42 (1.34)			
	Post2	11.8 (2.08)	11.6 (2.16)	11.1 (1.81)			
	ηp^2^				0.960	0.073	0.036
Standard bicarbonate (mmol/L) (SBC)	Pre	25.3 (0.99)	25.5 (1.90)	25.7 (1.32)	*p* ≤ 0.001	*p* = 0.251	*p* = 0.379
	Post1	21.3 (1.96)	21.1 (2.10)	21.7 (1.22)			
	Post2	16.5 (1.35)	16.7 (2.13)	17.6 (1.67)			
	ηp^2^				0.970	0.109	0.082
Bicarbonate anion (mmol/L) (HCO_3_^−^)	Pre	26.4 (1.35)	26.6 (2.51)	26.7 (1.71)	*p* ≤ 0.001	*p* = 0.497	*p* = 0.649
	Post1	21.7 (2.05)	21.6 (2.56)	22.3 (1.45)			
	Post2	16.6 (1.53)	16.8 (2.82)	17.8 (1.95)			
	ηp^2^				0.978	0.062	0.054
Actual base excess (mmol/L) (ABE)	Pre	1.11 (1.16)	1.44 (2.14)	1.54 (1.52)	*p* ≤ 0.001	*p* = 0.205	*p* = 0.249
	Post1	−3.77 (2.50)	−3.98 (2.66)	−3.23 (1.52)			
	Post2	−10.3 (2.02)	−10.4 (3.17)	−8.73 (2.37)			
	ηp^2^				0.962	0.124	0.104
Standard base excess (mmol/L) (SBE)	Pre	1.48 (1.37)	1.84 (2.61)	1.98 (1.80)	*p* ≤ 0.001	*p* = 0.246	*p* = 0.385
	Post1	−4.04 (2.64)	−4.22 (3.01)	−3.38 (1.67)			
	Post2	−10.9 (2.10)	−10.6 (3.48)	−9.26 (2.44)			
	ηp^2^				0.970	0.110	0.081
Glucose (mg/dL) (Glu)	Pre	99.8 (15.2)	103 (29.7)	101 (12.1)	*p* ≤ 0.001	*p* = 0.166	*p* = 0.258
	Post1	116 (13.0)	110 (17.5)	104 (16.0)			
	Post2	128.0 (16.1)	121.0 (17.1)	115 (18.2)			
	ηp^2^				0.462	0.139	0.103

*—significant difference (*p* ≤ 0.050) between 0% and 10%; ^#^—trend (*p* = 0.082) between 5% and 10%.

**Table 4:** Changes in biomarkers of electrolytes and osmolarity in capillary blood of the finger in pre-, post1 and post2 incremental test. Values are mean (SD).

		0%	5%	10%	Time	Condition	C × T
K^+^ (mmol/L)	Pre	4.76 (0.65)	4.95 (0.60)	5.04 (0.77)	*p* = 0.021	*p* = 0.641	*p* = 0.625
	Post1	5.19 (0.66)	4.93 (0.38)	5.18 (0.55)			
	Post2	5.34 (0.54)	5.21 (0.67)	5.33 (0.48)			
	ηp^2^				0.275	0.036	0.052
Na^+^ (mmol/L)	Pre	143 (1.19)	144 (2.27)	143 (1.30)	*p* ≤ 0.001	*p* = 0.280	*p* = 0.359
		143 (1.32)	144 (1.04)	144 (0.90)			
	Post	146 (1.72)	150 (1.9)	146 (1.60)			
	ηp^2^				0.791	0.101	0.085
Cl^−^ (mmol/L)	Pre	107 (1.33)	108 (3.10)	107 (2.43)	*p* ≤ 0.001	*p* = 0.868	*p* = 0.536
	Post1	108 (1.45)	108 (2.37)	108 (2.70)			
	Post2	109 (1.45)	109 (2.13)	109 (2.16)			
	ηp^2^				0.719	0.012	0.062
Ca^+^ (mmol/L)	Pre	1.27 (0.04)	1.28 (0.08)	1.26 (0.05)	*p* = 0.015	*p* = 0.779	*p* = 0.556
	Post1	1.24 (0.02)	1.24 (0.03)	1.24 (0.03)			
	Post2	1.27 (0.03)	1.27 (0.02)	1.27 (0.03)			
	ηp^2^				0.295	0.021	0.060
Anion_GAP (mmol/L)	Pre	9.38 (1.40)	9.21 (1.61)	9.01 (1.47)	*p* ≤ 0.001	*p* = 0.448	*p* = 0.274
	Post1	13.2 (2.17)	14.3 (1.83)	13.7 (1.57)			
	Post2	19.6 (2.41)	19.9 (2.44)	18.9 (1.87)			
	ηp^2^				0.959	0.065	0.099
mOsm (mmol/kg)	Pre	292 (1.97)	294 (3.94)	292 (2.46)	*p* ≤ 0.001	*p* = 0.566	*p* = 0.290
	Post1	293 (2.41)	294 (2.59)	294 (2.25)			
	Post2	299 (3.69)	299 (3.17)	298 (3.08)			
	ηp^2^				0.874	0.050	0.105

## Data Availability

The datasets used and/or analyzed during the current study are available from the corresponding author upon reasonable request.

## References

[1] de Waal S.J., Gomez-Ezeiza J., Venter R.E., Lamberts R.P. (2021). Physiological Indicators of Trail Running Performance: A Systematic Review. Int. J. Sports Physiol. Perform..

[2] Ehrström S., Tartaruga M.P., Easthope C.S., Brisswalter J., Morin J.B., Vercruyssen F. (2018). Short Trail Running Race: Beyond the Classic Model for Endurance Running Performance. Med. Sci. Sports Exerc..

[3] Costa R.J.S., Knechtle B., Tarnopolsky M., Hoffman M.D. (2019). Nutrition for Ultramarathon Running: Trail, Track, and Road. Int. J. Sport Nutr. Exerc. Metab..

[4] Silder A., Besier T., Delp S.L. (2015). Running with a load increases leg stiffness. J. Biomech..

[5] Busch A., Trounson K., Browne P., Robertson S. (2022). Effects of lower limb light-weight wearable resistance on running biomechanics. J. Biomech..

[6] Macadam P., Cronin J.B., Simperingham K.D. (2017). The Effects of Wearable Resistance Training on Metabolic, Kinematic and Kinetic Variables During Walking, Running, Sprint Running and Jumping: A Systematic Review. Sports Med..

[7] Lobb N.J., Fain A.C., Seymore K.D., Brown T.N. (2019). Sex and stride length impact leg stiffness and ground reaction forces when running with body borne load. J. Biomech..

[8] Purdom T.M., Mermier C., Dokladny K., Moriarty T., Lunsford L., Cole N., Johnson K., Kravitz L. (2021). Predictors of Fat Oxidation and Caloric Expenditure With and Without Weighted Vest Running. J. Strength. Cond. Res..

[9] Keren G., Epstein Y., Magazanik A., Sohar E. (1981). The energy cost of walking and running with and without a backpack load. Eur J. Appl. Physiol. Occup. Physiol..

[10] Jiménez-Redondo G., Castro-Frecha B., Martínez-Noguera F.J., Alcaraz P.E., Marín-Pagán C. (2024). Physiological Responses in Trail Runners during a Maximal Test with Different Weighted-Vest Loads. Sports.

[11] Urbaniak G.C., Plous S. (2013). Research Randomizer.

[12] World Medical Association (2013). World Medical Association Declaration of Helsinki: Ethical principles for medical research involving human subjects. JAMA.

[13] Stewart A., Marfell-Jones M., Olds T., De Ridder H. (2011). International Society for advancement of Kinanthropometry. International Standards for Anthropometric Assessment.

[14] Faulkner J.A. (1966). Physiology of swimming. Res. Q..

[15] Norton K., Norton K., Eston R. (2018). Standards for Anthropometry Assessment. Kinanthropometry and Exercise Physiology.

[16] Zuniga J.M., Housh T.J., Camic C.L., Bergstrom H.C., Traylor D.A., Schmidt R.J., Johnson G.O. (2012). Metabolic parameters for ramp versus step incremental cycle ergometer tests. Appl. Physiol. Nutr. Metab..

[17] Larson R., Cantrell G., Ade C., Farrell Iii J., Lantis D., Barton M., Laron D. (2015). Physiologic responses to two distinct maximal cardiorespiratory exercise protocols. Int. J. Sports Exerc. Med..

[18] Binder R.K., Wonisch M., Corra U., Cohen-Solal A., Vanhees L., Saner H., Schmid J.-P. (2008). Methodological approach to the first and second lactate threshold in incremental cardiopulmonary exercise testing. Eur. J. Prev. Cardiol..

[19] Scherr J., Wolfarth B., Christle J.W., Pressler A., Wagenpfeil S., Halle M. (2013). Associations between Borg’s rating of perceived exertion and physiological measures of exercise intensity. Eur. J. Appl. Physiol..

[20] Mier C.M., Alexander R.P., Mageean A.L. (2012). Achievement of VO2max criteria during a continuous graded exercise test and a verification stage performed by college athletes. J. Strength. Cond. Res..

[21] Zhang J.B., Lin J., Zhao X.D. (2015). Analysis of bias in measurements of potassium, sodium and hemoglobin by an emergency department-based blood gas analyzer relative to hospital laboratory autoanalyzer results. PLoS ONE.

[22] Cohen J. (2009). Statistical Power Analysis for the Behavioral Sciences.

[23] Cartón-Llorente A., Rubio-Peirotén A., Cardiel-Sánchez S., Roche-Seruendo L.E., Jaén-Carrillo D. (2023). Training Specificity in Trail Running: A Single-Arm Trial on the Influence of Weighted Vest on Power and Kinematics in Trained Trail Runners. Sensors.

[24] Birrell S.A., Haslam R.A. (2009). The effect of military load carriage on 3-D lower limb kinematics and spatiotemporal parameters. Ergonomics.

[25] Birrell S.A., Hooper R.H., Haslam R.A. (2007). The effect of military load carriage on ground reaction forces. Gait Posture.

[26] Kinoshita H. (1985). Effects of different loads and carrying systems on selected biomechanical parameters describing walking gait. Ergonomics.

[27] Harman E., Han K.H., Frykman P., Pandorf C. (2000). The Effects of Backpack Weight on the Biomechanics of Load Carriage.

[28] Polcyn A.F., Bensel C.K., Harman E.A., Obusek J.P., Pandorf C., Frykman P. (2002). Effects of Weight Carried by Soldiers: Combined Analysis of Four Studies on Maximal Performance, Physiology, and Biomechanics.

[29] Tilbury-Davis D.C., Hooper R.H. (1999). The kinetic and kinematic effects of increasing load carriage upon the lower limb. Hum. Mov. Sci..

[30] Wang H., Frame J., Ozimek E., Leib D., Dugan E.L. (2012). Influence of fatigue and load carriage on mechanical loading during walking. Mil. Med..

[31] Wang H., Frame J., Ozimek E., Leib D., Dugan E.L. (2013). The effects of load carriage and muscle fatigue on lower-extremity joint mechanics. Res. Q. Exerc. Sport.

[32] Verbitsky O., Mizrahi J., Voloshin A., Treiger J., Isakov E. (1998). Shock transmission and fatigue in human running. J. Appl. Biomech..

[33] Voloshin A.S., Mizrahi J., Verbitsky O., Isakov E. (1998). Dynamic loading on the human musculoskeletal system—Effect of fatigue. Clin. Biomech..

[34] Derrick T.R., Dereu D., McLean S.P. (2002). Impacts and kinematic adjustments during an exhaustive run. Med. Sci. Sports Exerc..

[35] Mizrahi J., Verbitsky O., Isakov E., Daily D. (2000). Effect of fatigue on leg kinematics and impact acceleration in long distance running. Hum. Mov. Sci..

[36] Christina K.A., White S.C., Gilchrist L.A. (2001). Effect of localized muscle fatigue on vertical ground reaction forces and ankle joint motion during running. Hum. Mov. Sci..

[37] James C.R., Dufek J.S., Bates B.T. (1994). 565 Fatigue Accommodation During Landing. Med. Sci. Sports Exerc..

[38] Puthoff M.L., Darter B.J., Nielsen D.H., Yack H.J. (2006). The effect of weighted vest walking on metabolic responses and ground reaction forces. Med. Sci. Sports Exerc..

[39] Gaffney C.J., Cunnington J., Rattley K., Wrench E., Dyche C., Bampouras T.M. (2022). Weighted vests in CrossFit increase physiological stress during walking and running without changes in spatiotemporal gait parameters. Ergonomics.

[40] Martínez-Noguera F.J., Alcaraz P.E., Carlos-Vivas J., Marín-Pagán C. (2022). Chronic Supplementation of 2S-Hesperidin Improves Acid-Base Status and Decreases Lactate at FatMax, at Ventilatory Threshold 1 and 2 and after an Incremental Test in Amateur Cyclists. Biology.

[41] Cannon D.T., Howe F.A., Whipp B.J., Ward S.A., McIntyre D.J., Ladroue C., Griffiths J.R., Kemp G.J., Rossiter H.B. (2013). Muscle metabolism and activation heterogeneity by combined ^31^P chemical shift and T_2_ imaging, and pulmonary O_2_ uptake during incremental knee-extensor exercise. J. Appl. Physiol. (1985).

[42] Copp S.W., Hirai D.M., Musch T.I., Poole D.C. (2010). Critical speed in the rat: Implications for hindlimb muscle blood flow distribution and fibre recruitment. J. Physiol..

[43] Hostrup M., Cairns S.P., Bangsbo J. (2011). Muscle Ionic Shifts During Exercise: Implications for Fatigue and Exercise Performance. Compr. Physiol..

[44] Hureau T.J., Broxterman R.M., Weavil J.C., Lewis M.T., Layec G., Amann M. (2022). On the role of skeletal muscle acidosis and inorganic phosphates as determinants of central and peripheral fatigue: A 31P-MRS study. J. Physiol..

[45] Moxnes J.F., Sandbakk Ø. (2012). The kinetics of lactate production and removal during whole-body exercise. Theor. Biol. Med. Model..

